# A Breakthrough Application of a Cross-Linked Polystyrene Anion-Exchange Membrane for a Hydrogencarbonate Ion-Selective Electrode

**DOI:** 10.3390/s19061268

**Published:** 2019-03-13

**Authors:** Sylwia Dabrowska, Jan Migdalski, Andrzej Lewenstam

**Affiliations:** Faculty of Materials Science and Ceramics, AGH—University of Science and Technology, Mickiewicza 30, 30-059 Krakow, Poland; sd.dabrowska@gmail.com (S.D.); migdal@agh.edu.pl (J.M.)

**Keywords:** bicarbonate ion-selective electrode, polystyrene matrix, anion exchange membrane, direct potentiometry, simplex optimization

## Abstract

Polystyrene cross-linked with divinylbenzene and functionalized by a quaternary ammonium cation anion site is used as the membrane of a hydrogencarbonate (i.e., bicarbonate) ion-selective electrode. The polystyrene matrix membrane improves the selectivity towards interfering lipophilic ions in comparison to previously described polyvinyl chloride membranes. The reason for this behaviour is sought in coupled ion-exchange and pore-diffusion processes in the membrane and the resulting kinetic discrimination of interfering ions. The electrode is successfully used for determination of bicarbonates in mineral drinking waters. The simplex method is employed to refine the analytical outcome.

## 1. Introduction

Hydrogencarbonate (bicarbonate) is the only anion which is not directly measured by ion-selective electrodes (ISEs), even at the mmol L^−1^ concentrations found in blood [1]. Direct sensitivity to bicarbonate ions has been occasionally reported previously, but it has finally been ascribed to hydroxyl [2] ions or carbonate ions [3,4,5,6] and not to bicarbonates. The fact is that, so far, the determination of bicarbonates is possible only by indirect methods, e.g., [3,7,8].

The primary reason for the difficulty in designing a bicarbonate-sensitive ion-selective electrode is in the native properties of the bicarbonate anion, i.e., its hydration energy and ionic potential. These properties both determine its place in the Hoffmeister series which is close to that of chlorides. This makes finding a membrane with sufficient selectivity, especially towards chloride ions frequently present in real samples, extremely difficult. Additionally, the direct influence of hydrogen ions and pH-dependent carbonate ions impose a severe challenge.

Another drawback stems from the routines used in ISE membrane technology. Typically, the polymer applied to support electroactive ISE membrane components is a plasticized polyvinyl chloride (PVC) [9]. Although its suitability is well proved, ongoing research aims to take advantage of other polymers to reduce or eliminate plasticizer leaching, to improve biocompatibility, to improve sensor performance, its response time, selectivity, and detection limit. For these reasons, many other polymers have been tested, namely viscose cellulose, silicone rubber, cellulose acetate, acrylic polymers and polyurethanes [4,6,10,11,12,13,14,15].

In our previous work [16], we proposed a bicarbonate electrode based on a PVC membrane containing quaternary ammonium bicarbonate as ion exchanger. It was shown that the change of plasticizer or even its elimination from the PVC membrane caused only a slight improvement in the selectivity. This report presents an application of a divinylbenzene cross-linked polystyrene anion ion-exchanger functionalized by quaternary ammonium bicarbonate as a membrane in an ion-selective bicarbonate electrode. We demonstrate that with use of such a membrane, a bicarbonate ISE with substantially improved analytical properties can be obtained. Although polystyrene anionic membranes have recently been used in fuel cells and electrodialysis [17,18], to the best of our knowledge the functionalization and application of this membrane type for a bicarbonate ISE is reported here for the first time.

## 2. Materials and Methods

### 2.1. Reagents

All the compounds used were purchased from Avantor Performance Materials (Gliwice, Poland) or Sigma-Aldrich (Darmstadt, Germany). Distilled and deionized water was used for preparation of the aqueous solutions. Natural mineral healing waters: Franciszek and Jozef (Wysowa, Poland), Jan and Slotwinka (Krynica Zdroj, Poland), Donat Mg (Rogaska Slatina, Slovenia) and mineral waters: Cisowianka (Naleczow, Poland), Kryniczanka (Krynica Zdroj, Poland), Staropolanka (Polanica Zdroj, Poland) were used. These waters were chosen because of the different total content of mineral electrolytes. Moreover, some of these waters contain relatively high concentrations of chloride ions and other interferents (SO_4_^2−^, Br^−^). Their composition specified by the manufacturer is shown in Table 1. In order to test potentiometric ion-selective electrodes with a cross-linked polystyrene anion-exchange membrane for the direct determination of bicarbonate ions, several samples (Samples 1–6, see Table 1) with a known concentration of bicarbonate and chloride ions were used.

### 2.2. Ion-Exchanger Membrane

A polystyrene anion-exchange membrane cross-linked with divinylbenzene and functionalized with quaternary ammonium chloride salt (AMI-7001S) was obtained from Membranes International Inc. (Ringwood, NJ, USA). The membrane thickness was 0.45 mm, total anion-exchange capacity 1.3 meq·g^−1^ and electrical resistance <40 Ω·cm^−2^.

### 2.3. Hydrogencarbonate (Bicarbonate) Ion-Selective Electrode

A diaphragm sheet (AMI-7001S, Membranes International Inc., Ringwood, NJ, USA) was used as a primary material to cut out the membrane rings of 7 mm diameter which were then placed in the electrode body of an IS-561 device (Philips, Sigma-Aldrich, Darmstadt, Germany). 10^−1^ mol L^−1^ NaHCO_3_ and 10^−4^ mol L^−1^ NaCl solution and silver chloride electrode form the internal contact. Before potentiometric measurements, the electrodes were conditioned (soaked) for at least 24 h in 10^−1^ mol L^−1^ NaHCO_3_ solution. During soaking, the solution was changed several times to ensure complete filling of the disposable cationic sites by hydrogencarbonate ions [16].

### 2.4. Potentiometric Measurements

Potentiometric measurements were carried out with a multichannel potentiometric meter, with an input resistance higher than 10^13^ Ω and an input current at the level 10^−15^ A, coupled with computer data collection and recording. The reference electrode was a double-junction reference Ag/AgCl electrode (Radiometer REF251, Loveland, CO, USA) with an external electrolyte 10^−1^ mol L^−1^ K_2_SO_4_. Potentiometric measurements were performed at room temperature (23–25 °C).

### 2.5. Measuring Procedure

The selectivity coefficient was determined by two methods for the following interfering ions: SO_4_^2−^ (Na_2_SO_4_), Cl^−^ (NaCl), Br^−^ (NaBr). In both methods, the semi-empirical Nikolskii-Eisenman equation:

(1) $$E=E^{0}+S\log(a_{i}+{\sum K_{i,j}^{pot}\cdot a_{j}}^{z_{i}/z_{j}})$$

is used for calculation of the selectivity coefficient $K_{i,j}^{pot}$ where *i* denotes a primary (main) ion and *j* an interfering ion. Prior to determination of the selectivity coefficients, the electrodes were conditioned overnight in 0.1 mol L^−1^ NaHCO_3_ solution. The selectivity coefficients were determined by the separate solution method according to the following formula, using the experimental slope:

(2) $$\log K_{HCO_{3}^{-},j}^{pot}=\left(E_{j}^{0}-E_{HCO_{3}^{-}}^{0}\right)/S_{HCO_{3}^{-}}$$

where $\log K_{HCO_{3}^{-},j}^{pot}$ is the logarithm of selectivity coefficient, $S_{HCO_{3}^{-}}$ is the slope of the bicarbonate electrode, and $E_{j}^{0}$ and $E_{HCO_{3}^{-}}^{0}$ are the values obtained by the extrapolated calibration curves for various anions and hydrogencarbonate to log a = 0.

In the separate solution method (SSM), the changes in the potentials were measured in NaHCO_3_ solution and then in the interferent solution within concentrations of 10^−5^–10^−1^ mol L^−1^. On the other hand, in the fixed primary ion method (FPM), the potentials were measured in the solutions with constant activity of NaHCO_3_ (NaHCO_3_ concentration was 25 mmol L^−1^) and varying interferent activities. The selectivity coefficient, $K_{HCO_{3}^{-},j}^{pot}$, was calculated from the following equation:

(3) $$K_{HCO_{3}^{-},j}^{pot}=a_{HCO_{3}^{-}}/a_{j}{}^{1/z_{j}}$$

where *a* is the ion activity and *z_j_* is the electrical charge of the interfering ion. The activity of the individual ions was calculated according to the Debye-Hückel equation.

### 2.6. Energy Dispersive X-ray Spectroscopy (EDS) and X-ray Photoelectron Spectroscopy (XPS)

The chemical composition of the membrane is monitored by Energy Dispersive X-ray Spectroscopy, EDS (LINK ISIS 300, Oxford Instruments, UK) and X-ray photoelectron spectroscopy, XPS (PHI 5000 VersaProbe, Physical Electronics, Inc., Chanhassen, MN, USA) with monochromatized Mg Kα line with energy 1253.6 eV). Studies were carried out with the membranes conditioned in 10^−1^ mol L^−1^ NaHCO_3_ and non-conditioned membranes.

## 3. Results and Discussion

### 3.1. Inducing Sensitivity towards Bicarbonates

The main idea of inducing bicarbonate sensitivity is to use an ion-exchanger membrane containing the sites so as to electrostatically attract anions. In our case, the sites are positively charged quaternary ammonium ions ($R_{4}N^{+}$) dispersed in the polymer bulk as well as on the surfaces of the pores inside the polymer membrane, charge-balanced by Cl^−^ ions. Our aim was to load the sites to the largest possible degree by the counter ions of our interest, bicarbonate ions (HCO_3_^−^). This was done by prolonged soaking the native anion-exchanger membrane in 0.1 mol L^−1^ solution of NaHCO_3,_ with the resulting ion-exchange:

$$R_{4}N^{+}Cl^{-}(m)+HCO_{3}^{-}(s)\overset{}{⇄}R_{4}N^{+}HCO_{3}^{-}(m)+Cl^{-}(s)$$

The exchange of native chlorides for bicarbonates is a necessary condition for making a membrane potentiometrically sensitive to that “main” ion, characterized by a slope typical for monovalent anions, where a theoretical value is: 59.2 mV at 25 °C.

**Figure 1:**
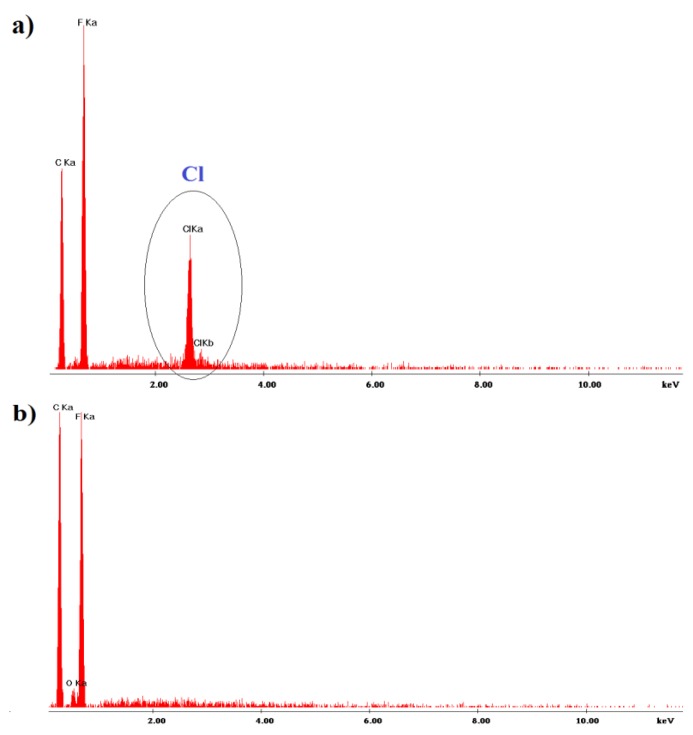
EDS spectra of the polystyrene membranes, (**a**) non-conditioned, (**b**) conditioned membrane in 0.1 mol L^−1^ NaHCO_3._

SEM/EDS analysis was conducted to investigate the change in the chemical composition of the membranes and the extent of substitution of the native chlorides to invited bicarbonates. Measurements were performed for a non-conditioned polystyrene membrane (Figure 1a) and for this membrane after conditioning in 10^−1^ mol L^−1^ NaHCO_3_ (Figure 1b). The EDS results indicate apparent replacement of chloride ions from lipophilic sites in the polystyrene membrane.

### 3.2. Sensitivity of the Sensor

**Figure 2:**
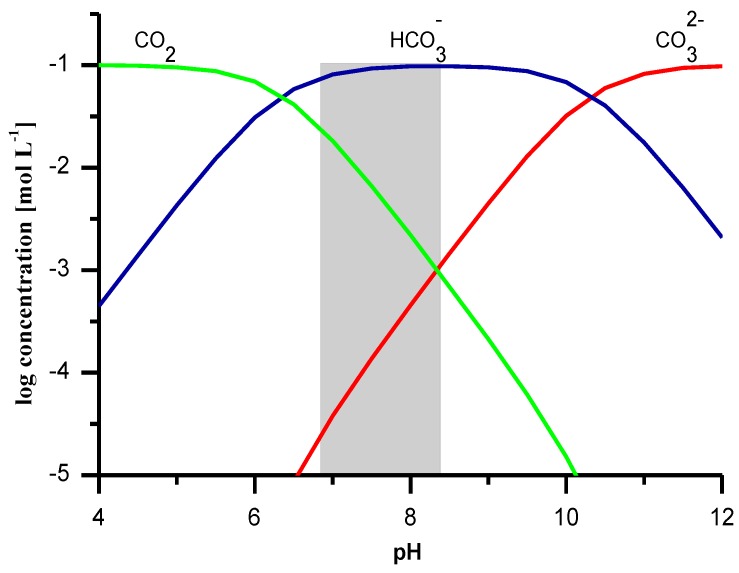
Forms of CO_2_ as a function of pH; total concentration of CO_2_ 0.1 mol L^−1^; temperature 25°C.

Presence of the main ion alone might not be sufficient for obtaining a bicarbonate ISE, because of the influence of the interfering ions which can also contribute to the ISE response by ion exchange. One great challenge comes naturally from carbonates. Since the soaking is done with the water, and the water saturates the membrane pores, we must consider that carbon dioxide exists in three different inorganic forms, bicarbonate (HCO_3_^−^), carbonate ions (CO_3_^2−^) and carbon dioxide (CO_2_), which appear depending on the pH (Figure 2).

The total carbonate species are related by the following equilibria:

$$CO_{2}(aq)+H_{2}O\overset{K_{1}}{⇄}HCO_{3}^{-}+H^{+}\overset{K_{2}}{⇄}CO_{3}^{2-}+2H^{+}$$

Chemical equilibria among these species in water are described by the constants which are related to activity:

(4) $$K_{1}=\frac{a_{HCO_{3}^{-}}\;a_{H^{+}}}{a_{CO_{2}}}=4.5\cdot 10^{-7}$$

(5) $$K_{2}=\frac{a_{CO_{3}^{2-}}\;a_{H^{+}}}{a_{HCO_{3}^{-}}}=4.7\cdot 10^{-11}$$

During soaking in 0.1 mol L^−1^ NaHCO_3_ in which the pH is 8.3, the bicarbonates are dominant but the concentration of carbonates in this solution is 0.001 mol L^−1^ and their influence should be considered. The carbonate ion may enter the membrane through the reaction:

$$2R_{4}N^{+}Cl^{-}(m)+CO_{3}^{2-}(s)\overset{}{⇄}{\left(R_{4}N^{+}\right)}_{2}CO_{3}^{2-}(m)+2Cl^{-}(s)$$

This process can affect the sensitivity of the received ion-selective electrodes and would be manifested by a decrease of the apparent slope, and in an extreme case to that characteristic for divalent ions, i.e., 29.6 mV.

**Figure 3:**
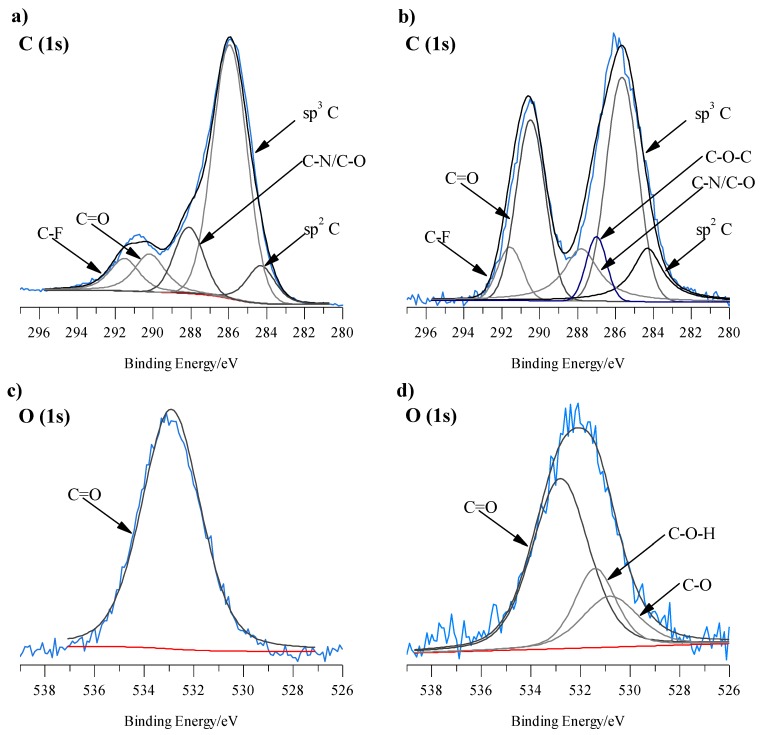
High-resolution XPS spectra of C(1s) and O(1s) recorded for non-conditioned membrane (**a,c**) and membrane conditioned in 0.1 mol L^−1^ NaHCO_3_ (**b,d**).

The presence of bicarbonates and carbonates in conditioned membranes was inspected by XPS. The soaking influenced C (1s) core level around 287 eV. After conditioned in NaHCO_3_ additional peak appear at ~286.95 eV confirming as shown in Figure 3. Interestingly, these peaks are escribed to C-O-C representing bicarbonates bonds (Figure 3b). Deconvolution of the high-resolution spectra of O (1s) peaks shows additional peaks at ~531.40 eV related to C-O-H and at ~530.65 eV related to C-O bonds that reconfirm the presence of bicarbonates. The obtained data indicate that the sensitivity of the ISE-HCO_3_^−^ electrodes is a mixed one and can be ascribed to both bicarbonate and carbonate ions.

In order to determine whether the sensitivity of the electrode with polystyrene anion-exchange membrane is directly dictated by the HCO_3_^−^ ion, in contrast to possible indirect mechanism by CO_3_^2^, a specially dedicated potentiometric measurement (*experimentum crucis*) was made. In accordance with the method described by Lewenstam et al. [19], the potential change ΔE of ion-selective electrodes was measured first in 25 mmol L^−1^ sodium bicarbonate solution and then in 25 mmol L^−1^ NaHCO_3_ solution mixed in 1/1 volume ratio with HEPES solution, 4-(2-hydroxyethyl)-1-piperazineethanesulfonic acid), at a concentration of 0.008 mol L^−1^. HEPES buffer solution addition, along with the change in pH, changes the concentration ratio of CO_3_^2−^/HCO_3_^−^ forms.

In the calculation of the theoretical potential change, ΔE, two cases are considered: when the electrode would show sensitivity only to carbonate ions:

(6) $$\Delta E_{CO_{3}^{2-}}=-2.303\frac{RT}{2F}\log\frac{a_{CO_{3}^{2-}(L)}}{a_{CO_{3}^{2-}(H)}}=38.2\;mV$$

or only to bicarbonate ions:

(7) $$\Delta E_{HCO_{3}^{-}}=-2.303\frac{RT}{F}\log\frac{a_{HCO_{3}^{-}(L)}}{a_{HCO_{3}^{-}(H)}}=19.1\;mV$$

$a_{\left(H\right)}$, $a_{\left(L\right)}$—is the activity of the ion in solution containing 25 mmol L^−1^ NaHCO_3_ and solution with HEPES respectively.

The ion activities were calculated from the following formulas:

(8) $$a_{HCO_{3}^{-}}=\frac{K_{1}C_{T}a_{H^{+}}}{{\left(a_{H^{+}}\right)}^{2}+K_{1}a_{H^{+}}+K_{1}K_{2}}$$

(9) $$a_{CO_{3}^{2-}}=\frac{K_{1}K_{2}C_{T}}{{\left(a_{H^{+}}\right)}^{2}+K_{1}a_{H^{+}}+K_{1}K_{2}}$$

where: K_1_ (pK_1_ = 6.35) and K_2_ (pK_2_ = 10.33)—dissociation constants of carbonic acid at a temperature of 25 °C [20], $a_{H^{+}}$—activity of the hydrogen ion, $C_{T}$—total concentration of NaHCO_3_. Activity coefficients were calculated using the Debye-Hückel equation. The measured pH values were used in the calculations.

The measured value of ΔE for the ion selective electrode with polystyrene membrane was 18.7 ± 0.2 mV. Comparing this experimentally determined ΔE with the theoretical prediction, it is evident that the electrodes exhibit sensitivity to bicarbonate ions. The charge transfer and membrane transport of these ions determine the response of the electrode, not the carbonate ions. Only under such circumstances, can bicarbonates be the main ions.

### 3.3. Electrode Response and Selectivity

**Figure 4:**
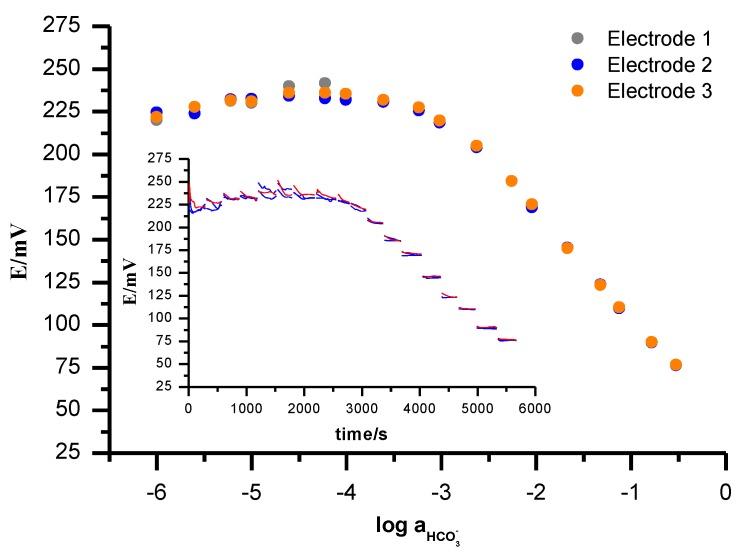
Potentiometric calibration curve and potential change over time (insert) of bicarbonate sensitive electrodes (n = 3) with a polymer matrix of divinylbenzene cross-linked polystyrene obtained during measurements in NaHCO_3_ solutions in the concentration range 10^−6^–5·10^−1^ mol L^−1.^

To check the response of the electrodes the calibrations in 10^−6^–5·10^−1^ mol L^−1^ of NaHCO_3_ were performed. A close-to-Nernstian response slope of -58 ± 2 mV/$\log a_{HCO_{3}^{-}}^{}$ was found for bicarbonate in the linear part of the calibration curve (5·10^−4^–5·10^−1^ mol L^−1^). The value of detection limit was (1.4 ± 0.3)·10^−4^ mol L^−1^. The limit of detection was determined from the intersection of the two linear segments of the calibration plot. The response time is shown in the insert in Figure 4. Each measurement was performed with three replicate electrodes and the average values with standard deviations were reported.

**Figure 5:**
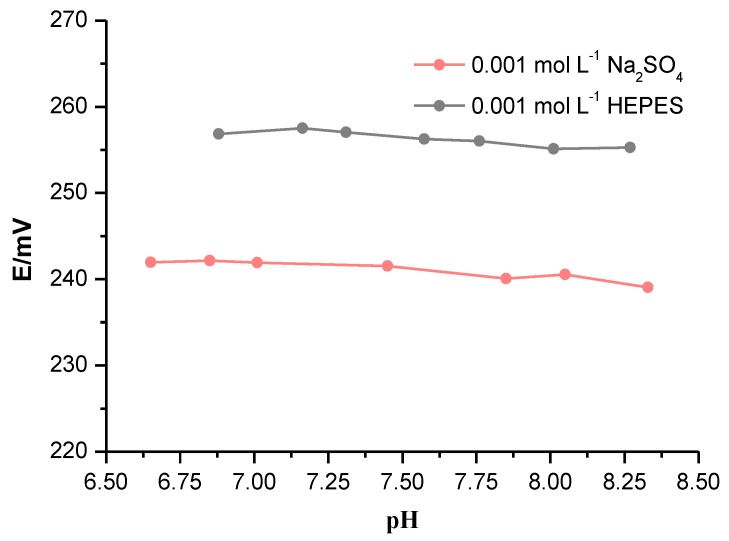
Effect of pH on the response of the potentiometric bicarbonate electrodes. The measurements were performed in HEPES buffer solution or Na_2_SO_4_. NaOH was used to change the pH.

Short-term stability can be obtained from Figure 4. The drift *E* over time is 0.1 mV min^−1^ for the polystyrene anion exchanger membrane. During calibration, a fast stabilization time of the signal was observed after the change of the main ion concentration; the response time t_90_ is less than 5 s. These very good dynamic properties of the bicarbonate electrode are crucial for analytical application as described later below. The lifetime of this electrode was about 2 months. During this time, the slope of the bicarbonate-sensitive electrode remained approximately stable. The effect of pH on electrode response in the pH range from 6.9 to 8.2 was purposely investigated. This range was dictated by practical application since it covers the pH range in the samples we used. The potential changes were measured in (a) 0.001 mol L^−1^ Na_2_SO_4_, (b) HEPES solutions at 0.001 mol L^−1^. The pH was changed by adding NaOH solution. The electrodes with a polystyrene membrane are not sensitive to pH change, as shown in Figure 5.

The selectivity coefficients measured by the two methods (SSM and FMP) and comparison of the selectivity coefficient determined by the SSM method of hydrogencarbonate ion-selective electrodes with a different polymer diaphragm matrix are presented in Table 2.

Similar selectivity coefficients were determined by both the SSM and the FPM methods. Table 2 compares the SSM selectivity coefficients for both the presently used and PVC membrane [16]. The influence of strongly interfering lipophilic chloride and bromide ions as well as divalent sulphides is, in the case of the ion-exchange membrane, much weaker than in the case of the polymeric PVC-based membranes reported before [16]. This observation is of crucial importance in the field of ISE technology.

The better selectivity for strongly interfering lipophilic anions, in our case chlorides and bromides vs. bicarbonates, can be ascribed to the chemical properties and the structure of the anion-exchange membrane used. The membrane is characterized by high ion-exchange capacity, and its 3D structure is made of nanopore/capillary channels “dispersed” in a bulk phase [17,18,21,22,23]. The transport of ions in such membranes can occur through solvent-filled nanopores/capillaries and the passive partition/diffusion of ions in the polystyrene phase. Immobile quaternary cation sites, available for counter ions (e.g., HCO_3_^−^), are present in a high number in the membrane. The ion-exchange process, initiated after contact of the membrane with bathing solution, spreads into the membrane bulk. The overall membrane selectivity results from a synergistic/collective effect of the ions permeating the membrane (through the channels and due to ion-partition in the polystyrene matrix) and the coulombic interaction between quaternary ammonium sites at the surface and in the membrane phase. The interfering chloride ions invited from a bathing solution have to substitute the main bicarbonate ions present in the membrane to exert an interfering effect. This happens in the course of a dynamic, time-dependent equilibration process, which influences the selectivity coefficients measured. The mechanism is theoretically interpreted by a diffusion layer model (DLM), which was introduced [24] and reviewed more recently by Lewenstam [25].

In general, the DLM states that the selectivity of the membranes’$K_{i,j}^{pot}$ variability is manifested in the range set by transport parameters (*kinetic discrimination*) and thermodynamic parameters (*true, unbiased selectivity*). The DLM predicts that kinetic discrimination effects should be pronounced for the membranes with high ion-exchange capacity, equivalent to high site number [26], in particular for anion-exchange membranes [27]. According to the DLM, the selectivity coefficient in our case is represented by the ratio of transport parameters, e.g., ion mobilities or diffusion coefficients of the interfering to main ion and is $\frac{D_{Cl^{´-}}}{D_{HCO_{3}^{-}}}=1.7$. Indeed, as shown below, a very close value $K_{HCO_{3}^{-},Cl^{-}}^{pot}\approx 1.3$ was determined, which indicates that a strong ion interference is indeed suppressed by kinetic discrimination. The true (thermodynamic) selectivity, given by a product of ion mobility ratio and ion-exchange constant (ion permeation ratio), is of 10^1^–10^2^ times higher [28,29]. The properties of the polystyrene matrix favour the kinetic discrimination process in comparison to PVC membranes.

### 3.4. Determination of Bicarbonates in Samples by Simplex Method

The membrane studied in our present research allowed taking advantage of concurrently improved selectivity via: (1) a new membrane matrix and (2) the kinetic discrimination effect.

A kinetic discrimination by definition necessitates optimizing the measurement time to get the best analytical results. Herein the optimization is achieved by the simplex method [30]. The principle of the simplex optimization is the displacement of an initial design (geometric figure) through the region studied in order to avoid experimental regions with undesirable responses. The initial geometric figure comprises *k* + 1 vertex, where *k* equals the number of variables in a *k*-dimensional domain. Therefore, the simplex in one dimensional (for one variable) is represented by a line and in three dimensional, by the tetrahedron (three variables). The simplex displacement is carried out by the reflection of the experimental point showing the worst response generating a new simplex that should be once again analyzed and displaced to the optimal region.

**Figure 6:**
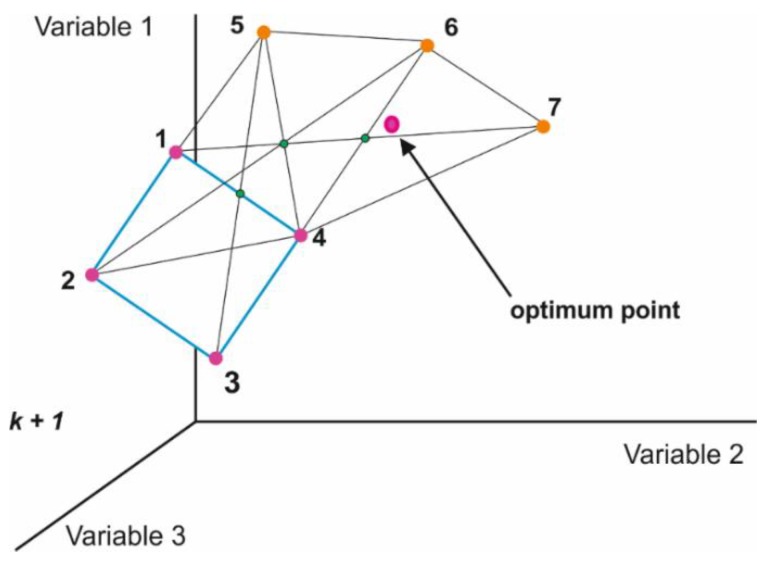
Example of the displacement of initial simplex to the region of optimum response of three variables.

**Figure 7:**
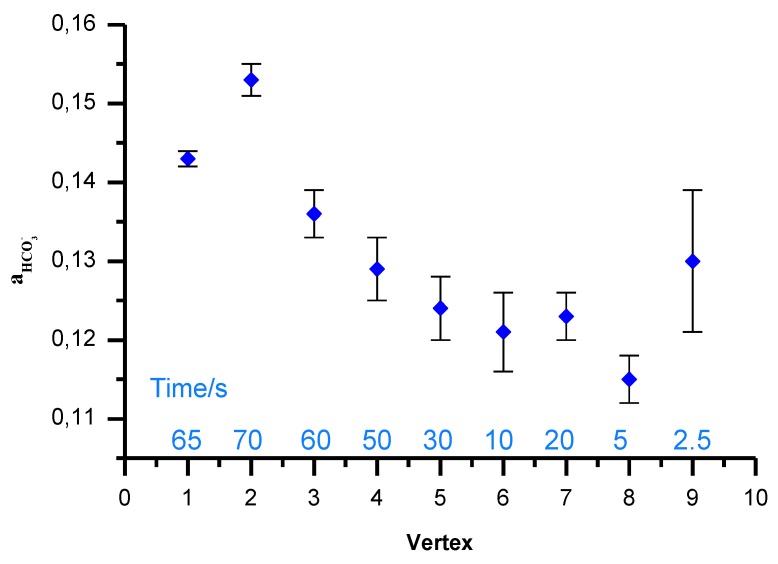
Minimizing the influence of chloride ion by the optimization of readout time in simplex measurements; the sample contains 25 mmol L^−1^ NaHCO_3_ and 0.1 mol L^−1^ NaCl. The bars represent standard deviation for three identical electrodes.

Figure 6 shows the simplex method with regard to the optimization of three variables through the displacement of the initial simplex represented by the geometric figure. First, in order to optimize the measurement time, the calibration was carried out in sodium bicarbonate solutions and then in the sample containing interfering ions (chlorides) at different time periods, starting at 65 s for a readout time and decreasing to 2.5 s. Calibration was carried out in solutions with the following concentrations: 10^−3^, 5 × 10^−3^, 10^−2^, 5 × 10^−2^, 10^−1^ mol L^−1^ NaHCO_3_, and in the sample of 25 mmol L^−1^ NaHCO_3_ and 0.1 mol L^−1^ NaCl which was used to estimate deviation from the target bicarbonate sample concentration, 25 mmol L^−1^ and corresponding activity 19.5. The measured apparent activity of bicarbonate ions in the sample was calculated using Equation (1) for S = 58,3 mV/$\log a_{HCO_{3}^{-}}^{}$ and K = 3. Although still far the target the closest, and reproducible (n = 3) readout was found after 5 s (Figure 7). Error bars are standard deviations obtained for measurements with three bicarbonate ion-selective electrodes.

Obviously in such a case, the discrimination of interfering chloride ions is due to kinetic discrimination.

After the optimal time of measurement time was found, multi-dimensional calibration and simplex optimization were performed.

The response of the ion selective electrode to primary ion (bicarbonate) and interferents (chloride ion) is described by the Nikolskii-Eisenman equation:

(10) $$E=E^{0}+S\log(a_{HCO_{3}^{-}}+K_{HCO_{3}^{-},Cl^{-}}^{pot}\cdot a_{Cl^{-}})$$

where *E^0^* is the standard potential, *S* is the slope of the linear part of the calibration curve, $K_{HCO_{3}^{-},Cl^{-}}^{pot}$ is the selectivity coefficient.

The reason for applying of the multi-dimensional calibration and simplex optimization is in the apparent interdependence of selectivity, formal potential, and slope in the Nikolskii-Eisenman equation. In the presence of interferents the slope of the characteristics of the bicarbonate electrode *S* and the value of *E^0^* may change, which can lead to significant errors in the analysis that need to be circumvented. In conventional potentiometry with ISEs the value of *E^0^ S*, and selectivity coefficients are determined prior to the sample measurements and assumed to be applicable in sample measurements. Such methodology presents the risk of inadequate use of the Nikolskii-Eisenman equation parameters and error in determination of the sample analyte. The simplex method allows concurrent optimizing the selectivity coefficient $K_{HCO_{3}^{-},Cl^{-}}^{pot}$, the slope *S* and *E^0^* in the target Equation (10).

The measurements were thus carried out at three concentration levels of HCO_3_^−^ and Cl^−^ in the range of 10^−3^–10^−1^ mol L^−1^ in two measuring series (solutions 1.1–1.3 and 2.1–2.3). The concentration of HCO_3_^−^ and Cl^−^ ions in the calibration solutions are summarized in Table 3. Three identical ion-selective bicarbonate electrodes were used for the measurements; the measurement time in each solution was 5 s. In all measuring series, the measurements were repeated five times.

Next, the slope values and *E^0^* obtained during calibration in NaHCO_3_ solutions without the presence of interferents and the value of the selectivity coefficient obtained by SSM were used to determine the initial vertices. Calculations of the initial values of the vertexes are presented in Table 4.

The simplex routine varies the values of the selectivity coefficient $K_{HCO_{3}^{-},Cl^{-}}^{pot}$, standard cell potential E^0^ and slope according to the rules of the simplex algorithm until the error depicted by Equation (11) is as low as possible:

(11) $$err=\sqrt{\sum {\left(a_{k}^{*}-a_{k}\right)}^{2}/a_{k}^{2}}/n$$

In this equation, $a_{k}^{*}$ is the designated activity of HCO_3_^−^, $a_{k}$ is the theoretical activity of the ion, while *n* is the number of solutions in a given series of measurements (n = 3). A Microsoft Excel spreadsheet was used for the calculations.

The optimized parameter values from the two measurements series which ensure the best fit between the determined main ion activity from the Nikolskii-Eisenman equation and the known target value of this ion are, respectively: *E^0^* = 26.69 ± 1.69 mV, *S* = −58.77 ± 0.28 mV/log a HCO_3_^−^ and $K_{HCO_{3}^{-},Cl^{-}}^{pot}$ = 1.31 ± 0.02.

To prove the validity of this approach we determined the concentrations of HCO_3_^−^ ions in samples with a known concentration of anions, and then in the real samples of mineral water. Three identical ion-selective bicarbonate electrodes were used for the measurements; the measurements were repeated three times (n = 3). To determine the concentration in the real sample, potentiometric titration in each sample was additionally performed. The water samples were titrated with 10^−1^ mol L^−1^ HCl solution until pH 4 was attained. The obtained results with standard deviation values and relative errors are summarized in Table 5. Relative error is calculated as the difference between the concentration measured by bicarbonate ion-selective electrode and the true concentration (the values of concentration given in Table 1) divided by the true concentration.

Optimization of the parameters in the Nikolskii-Eisenman equation allows satisfactory determination of hydrogencarbonate ions in the test sample, even in the presence of interferents at high concentrations. It is possible to determine the concentration of bicarbonate ions in the samples without the re-calculation of parameters from the Nikolskii-Eisenman equation for 10 days. After 20 days of conditioning, the relative error for all samples was about 40%.

The simplex method allowed minimizing the influence of the matrix. In the conventional case while determining the analyte concentration with the sample, with the *E^0^* and *S* values derived from the calibration curve and the $K_{HCO_{3}^{-},Cl^{-}}^{pot}$ determined by the SSM method, and without optimized time the analyte concentrations are significantly overestimated (Table 5, column named N-E equation). For example, for sample No. 2 where the concentration of bicarbonates is 5 times higher than that of interferent ions, the relative error would be +29.9% bigger, while for sample No. 6 where the interferent of the concentration is four times higher, the error would be as high as +326.2%.

Notwithstanding, both the theoretical and analytical approaches presented herein need to be further advanced. The limitation of this method is selectivity, especially for samples with the high content of interfering ions. The goal lies in finding even better membrane materials, an interpretation that would widen the transport concept (i.e., the influence of membrane porosity and tortuosity), measurement strategies and adequate analytical applications, which comprise the driving force of our ongoing research.

## 4. Conclusions

Hydrophilic anionic membrane made of divinylbenzene cross-linked polystyrene with high ion-exchange capacity is a promising material for designing a bicarbonate ion-selective electrode. The electrodes used showed a stable and fast response but much better selectivity in comparison to similar PVC-based membranes. The lesser influence of lipophilic ions can be attributed to the kinetic discrimination and a high ion-exchange capacity of the membranes used. Additionally, the membranes are insensitive to pH over a wide range.

The simplex method was satisfactorily employed to minimize the interference of chloride ions on the potentiometric signal. Optimization of the measurement time and parameters such as slope, standard potential and selectivity were achieved by application of the simplex method.

The use of an appropriate polymer matrix and selection of response parameters of the electrodes made possible the determination of the hydrogencarbonate ions in the presence of chloride ions even at high concentration.

We foresee that the spreading use of novel composite and heterogenous ion-exchange electrode materials and nano-porous membranes will widen the scope of application for potentiometric sensors. Moreover, by delivering further proof of the kinetic effects in the membrane formation process, the paradigm of ion-selective electrode membranes which are treated as a single phase able to create phase boundary potential will be revised.

## Figures and Tables

**Table 1:** Composition of mineral healing waters and mineral water samples from labels of the bottles.

**Mineral Water**					
**Water Sample**	Total mineral content [mg L^−1^]	HCO_3_^−^ [mmol L^−1^]	Cl^−^ [mmol L^−1^]	Br^−^ [mmol L^−1^]	SO_4_^2−^ [mmol L^−1^]
**Jan**	821.5	8.6	1.2	-	0.52
**Jozef**	2276.0	21.5	7.5	-	-
**Slotwinka**	3931.2	49.1	0.45	-	0.04
**Donat Mg**	13055.0	121.9	1.7	-	21.4
**Franciszek**	16030.0	139.0	65.0	11.2	11.2
**Cisowianka**	742.0	8.8	-	-	-
**Staropolanka**	800.0	9.3	0.19	-	0.33
**Kryniczanka**	2094.9	25.5	0.27	-	0.03
**Samples**					
	NaHCO_3_ [mmol L^−^^1^]		NaCl [mmol L^−1^]		
**1**	100		10		
**2**	50		10		
**3**	25		10		
**4**	100		100		
**5**	50		100		
**6**	25		100		

**Table 2:** Potentiometric selectivity coefficients determined by FPM and SSM method.

	Polystyrene Membrane		PVC Membrane
*Interfering ion*	$\log K_{HCO_{3}^{-},j}^{pot}$		$\log K_{HCO_{3}^{-},j}^{pot}$
	FPM	SSM	SSM
${\mathrm{SO}}_{4}^{2-}$	−0.44 ± 0.01	−0.33 ± 0.08	0.72 ± 0.05
${\mathrm{Cl}}^{-}$	0.65 ± 0.01	0.48 ± 0.01	1.08 ± 0.02
${\mathrm{Br}}^{-}$	0.65 ± 0.01	0.60 ± 0.05	2.45 ± 0.02

**Table 3:** Concentrations of ions in calibration solutions.

Series 1			Series 2		
No	HCO_3_^−^ [mol L^−1^]	Cl^−^ [mol L^−1^]	No	HCO_3_^−^ [mol L^−1^]	Cl^−^ [mol L^−1^]
1.1	10^−1^	10^−3^	**2.1**	10^−3^	10^−1^
1.2	10^−2^	10^−2^	**2.2**	10^−2^	10^−2^
1.3	10^−3^	10^−1^	**2.3**	10^−1^	10^−3^

**Table 4:** The values of initial vertices of the simplex optimization.

Vertex	E^0^ [mV]	S [mV/log a]	$K_{{\mathrm{HCO}}_{3}^{-}{,\mathrm{Cl}}^{-}}^{\mathrm{pot}}$
1	29.3	−58.1	3.02
2	29.3 + 1 · 0.1	−58.1	3.02
3	29.3 + 0.5 · 0.1	−58.1 + 0.87 · 0.1	3.02
4	29.3 + 0.5 · 0.1	−58.1 + 0.29 · 0.1	3.02 + 0.82 · 0.1

**Table 5:** Results of determination of bicarbonate ions.

Sample	Determined Bicarbonate [mmol L^−1^]	Relative Error* [%]	Determined Bicarbonate [mmol L^−1^]	Relative Error* [%]	N-E Equation Relative error [%]	Potentiometric Titration [mmol L^−1^]
	**Simplex, 1 day**		**Simplex, 10 days**			-
1	100.7 ± 3.3	0.7	97.7 ± 4.0	2.3	10.8	
2	49.6 ± 4.7	0.8	50.7 ± 3.8	1.4	29.9	
3	25.9 ± 0.7	3.6	25.5 ± 0.1	2.0	59.0	
4	100.6 ± 1.3	0.6	102.2 ± 5.7	2.2	156.5	
5	49.3 ± 5.2	1.4	50.9 ± 4.6	1.8	217.9	
6	23.9 ± 4.5	4.4	22.6 ± 3.0	9.6	326.2	
Jan	8.5 ± 0.9	1.2	8.7 ± 1.1	1.2	20.2	8.3 ± 0.3
Jozef	21.0 ± 3.2	2.3	21.4 ± 2.8	0.5	57.5	20.5 ± 1.4
Slotwinka	48.2 ± 5.6	1.8	47.1 ± 0.9	4.1	5.4	46.0 ± 0.7
Donat Mg	123.0 ± 7.5	0.9	123.7 ± 0.6	1.5	10.2	122.5 ± 3.5
Franciszek	142.6 ± 0.8	2.6	143.8 ± 2.5	3.5	66.4	139.8 ± 3.2
Cisowianka	8.9 ± 0.7	1.1	8.8 ± 0.5	0.0	6.9	8.8 ± 0.4
Staropolanka	10.1 ± 1.3	8.6	10.8 ± 0.7	16.1	19.8	10.5 ± 0.2
Kryniczanka	24.5 ± 0.2	3.9	23.8 ± 0.1	6.7	1.4	24.9 ± 2.1

* absolute number
